# Telomerase activity is required for the telomere G-overhang structure in *Trypanosoma brucei*

**DOI:** 10.1038/s41598-017-16182-y

**Published:** 2017-11-22

**Authors:** Ranjodh Sandhu, Bibo Li

**Affiliations:** 1 Center for Gene Regulation in Health and Disease, Department of Biological, Geological, and Environmental Sciences, Cleveland State University, 2121 Euclid Avenue, Cleveland, OH 44115 USA; 2 The Rockefeller University, 1230 York Avenue, New York, NY 10065 USA; 3 Department of Immunology, Lerner Research Institute, Cleveland Clinic, 9500 Euclid Avenue, Cleveland, OH 44195 USA; 4 Case Comprehensive Cancer Center, Case Western Reserve University, 10900 Euclid Avenue, Cleveland, OH 44106 USA; 5 Present Address: Department of Microbiology and Molecular Genetics, University of California Davis, One Shields Avenue, Davis, CA 95616 USA

## Abstract

*Trypanosoma brucei* causes fatal human African trypanosomiasis and evades the host immune response by regularly switching its major surface antigen, VSG, which is expressed exclusively from subtelomeric loci. Telomere length and telomere proteins play important roles in regulating VSG silencing and switching. *T*. *brucei* telomerase plays a key role in maintaining telomere length, and *T*. *brucei* telomeres terminate in a single-stranded 3′ G-rich overhang. Understanding the detailed structure of the telomere G-overhang and its maintenance will contribute greatly to better understanding telomere maintenance mechanisms. Using an optimized adaptor ligation assay, we found that most *T*. *brucei* telomere G-overhangs end in 5′ TTAGGG 3′, while a small portion of G-overhangs end in 5′ TAGGGT 3′. Additionally, the protein and the RNA components of the telomerase (*Tb*TERT and *Tb*TR) and *Tb*Ku are required for telomere G-overhangs that end in 5′ TTAGGG 3′ but do not significantly affect the 5′ TAGGGT 3′-ending overhangs, indicating that telomerase-mediated telomere synthesis is important for the telomere G-overhang structure. Furthermore, using telomere oligo ligation-mediated PCR, we showed for the first time that the *T*. *brucei* telomere 5′ end sequence – an important feature of the telomere terminal structure – is not random but preferentially 5′ CCTAAC 3′.

## Introduction

Telomeres are DNA/protein complexes located at chromosome ends. The specialized telomere structure masks the natural chromosome ends, preventing them from being recognized as DNA breaks by the DNA damage repair machinery; hence it is essential for maintaining chromosome stability^1^.

In most eukaryotic cells, telomere DNA consists of simple repetitive TG-rich sequences^2^ with a single-stranded G-rich 3′ overhang at the very end^3^. The overhang is essential for both telomere length maintenance and telomere end protection. Because conventional DNA polymerase cannot fully replicate the 5′ end of linear DNA molecules, telomeres shorten with each round of DNA replication^4^. Telomerase, a specialized reverse transcriptase, can synthesize telomere DNA *de novo* and counteract this “end replication problem”^5^. The telomere G-overhang usually serves as the DNA substrate for telomerase-mediated telomere elongation^3^. Additionally, proteins that bind to single-stranded telomere DNA can either stimulate telomerase activity (such as TPP1^6^) or prevent telomerase from accessing the telomere end (such as the CST complex^7^). Furthermore, long telomere G-overhangs can form a G-quadruplex structure that prevents telomerase binding and telomere elongation^8,9^. Finally, the 3′ telomere overhang can invade the duplex telomere region and form a T-loop structure, which has been identified in mammalian cells^10,11^, the hypotrichous ciliated protozoan *Oxytricha fallax* ^12^, and the protozoan parasite *Trypanosoma brucei* ^13^. The T-loop structure tucks away the 3′ G-overhang and presumably helps mask the chromosome ends from DNA damage repair machinery. Invasion of the overhang into the duplex telomere DNA on other chromosome ends also allow homologous recombination between different telomeres, which is important for telomerase-independent telomere maintenance^14,15^.

Telomere synthesis by telomerase is the predominant telomere elongation mechanism in *Trypanosoma brucei* ^16,17^, and the telomere G-overhang is presumably used as the primer substrate. Therefore, the *T*. *brucei* G-overhang structure is essential for telomere functions. The detailed structure of the *T*. *brucei* telomere G-overhang is unclear, although some telomeres have been reported to have a terminal sequence of 5′ TTAGGG 3′^18,19^. *T*. *brucei* is a protozoan parasite that causes fatal human African trypanosomiasis and regularly switches its major surface antigen, VSG, to evade the host immune response^20^. VSGs are exclusively expressed from VSG expression sites (ESs) located immediately upstream of the telomere repeats^21,22^, and DNA recombination is an important pathway for VSG switching^23–30^. Presumably, invasion of the telomere G-overhang into the duplex telomere region can initiate telomere recombination and influence recombination-mediated VSG switching, as ES-linked *VSGs* are located within 2 kb from the telomere repeats^22^ and telomere length and telomere proteins both influence VSG switching frequency^27–31^.

Telomere G-overhang length depends on multiple factors. Telomerase activity is expected to be an important determinant. Indeed, expression of telomerase results in longer G-overhangs at the leading daughter telomeres in human cells^32^ while dysfunctional telomerase leads to shorter telomere G-overhangs in tobacco and *S*. *castellii* ^33,34^. However, telomerase deletion does not affect telomere G-overhang length in *S*. *cerevisiae* ^35^, *Silene* plant seeds and leaves^36^, or mouse cells^37^, suggesting that telomere elongation by telomerase is not the only determinant of telomere G-overhang length in these organisms. Processing of the telomere 5′ end affects G-overhang length in some organisms. In mammalian cells, the 5′ end is resected by ExoI at both leading and lagging strands and by Apollo at the leading strand^38,39^. In budding yeast, 5′ telomere ends are mostly processed by Sae2/MRX, while ExoI and Sgs1 provide compensatory activities in the absence of Sae2/MRX^40^. In addition, removal of the most 5′ end primer during lagging strand DNA replication leaves a 3′ overhang, while the annealing position of this primer affects the overhang length. In human cells that often have 150–400 nt telomere G-overhang, the last RNA primer appears to be located 70–100 nt from the template end^41^. Outside the S phase in *S*. *cerevisiae*, the telomere G-overhang is only 12–14 nt long^42^, a length similar to that of the RNA primer used during lagging-strand synthesis^43^, suggesting that the last primer is annealed very close to the 3′ end of the template. Finally, C-strand fill-in synthesis by DNA polymerase – promoted by the CST complex^44^ – shortens the G-overhang.

The Ku70/80 dimer binds DNA ends and is essential for Non-Homologous End-Joining (NHEJ), one of the major DNA damage repair mechanisms^45^. A key function of Ku in NHEJ is to inhibit resection of the 5′ end at the DNA double strand break (DSB) site^45^. This is consistent with the observation that in *S*. *cerevisiae*, loss of Ku results in excessively long telomere G-overhangs throughout the cell cycle^46^. Additionally, loss of Ku results in a decrease in telomere length in budding yeast^47,48^, and depletion of Ku in human cells also causes telomere shortening^49^, indicating that Ku is important for telomere length maintenance. *T*. *brucei* lacks a homologue of DNA Ligase IV, a factor critical for NHEJ, and NHEJ events have not been observed to date. Still, *T*. *brucei* has Ku70/80 homologues, and loss of *Tb*Ku results in the same telomere shortening phenotype seen in telomerase null cells^50^. Therefore, although NHEJ may not occur in *T*. *brucei*, *Tb*Ku is essential for telomere length maintenance as its human and yeast homologues.

We have identified *Tb*TRF as a duplex telomere DNA binding factor in *T*. *brucei* ^51^. *Tb*TRF is essential for the telomere G-overhang structure^51^, but it is unclear whether other factors play important roles in telomere end processing. Here we used an optimized adaptor ligation assay to show that most *T*. *brucei* telomere 3′ ends have the 5′ TTAGGG 3′ sequence while a small portion of the 3′ ends have the 5′ TAGGGT 3′ sequence. We showed that the telomerase activity and *Tb*Ku are essential for the 5′ TTAGGG 3′ ends but does not affect the 5′ TAGGGT 3′ ends significantly. Our results clearly indicate that telomerase-mediated telomere elongation is a key factor determining the normal telomere G-overhang terminal sequences in *T*. *brucei*. In addition, using a Telomere Oligo Ligation-mediated PCR (TOLP) assay, we also determined the preferred last residues at the 5′ telomere end, which is an important feature of the telomere terminal structure and has not been determined in *T*. *brucei* previously.

## Results

### Most *T. brucei* telomere G-overhangs have a 3′ terminal sequence of 5′ TTAGGG 3′

Using native in-gel hybridization^52^, we and others have shown that *T*. *brucei* telomeres have single-stranded G-rich telomere overhangs^50,51^. However, only when undigested chromosomes are separated by Pulsed-Field Gel Electrophoresis (PFGE) can the telomere G-overhang be easily detected. The telomere G-overhang is clearly observed on minichromosomes in this assay, as *T*. *brucei* has ~ 100 minichromosomes that all migrate to approximately the same position in PFGE, therefore the signal is more concentrated. To examine more details of the telomere G-overhang structure, we adopted an adaptor ligation-mediated extension assay^53^ and confirmed that *T*. *brucei* telomeres indeed carry a G-overhang structure that is likely very short^18^.

**Figure 1:**
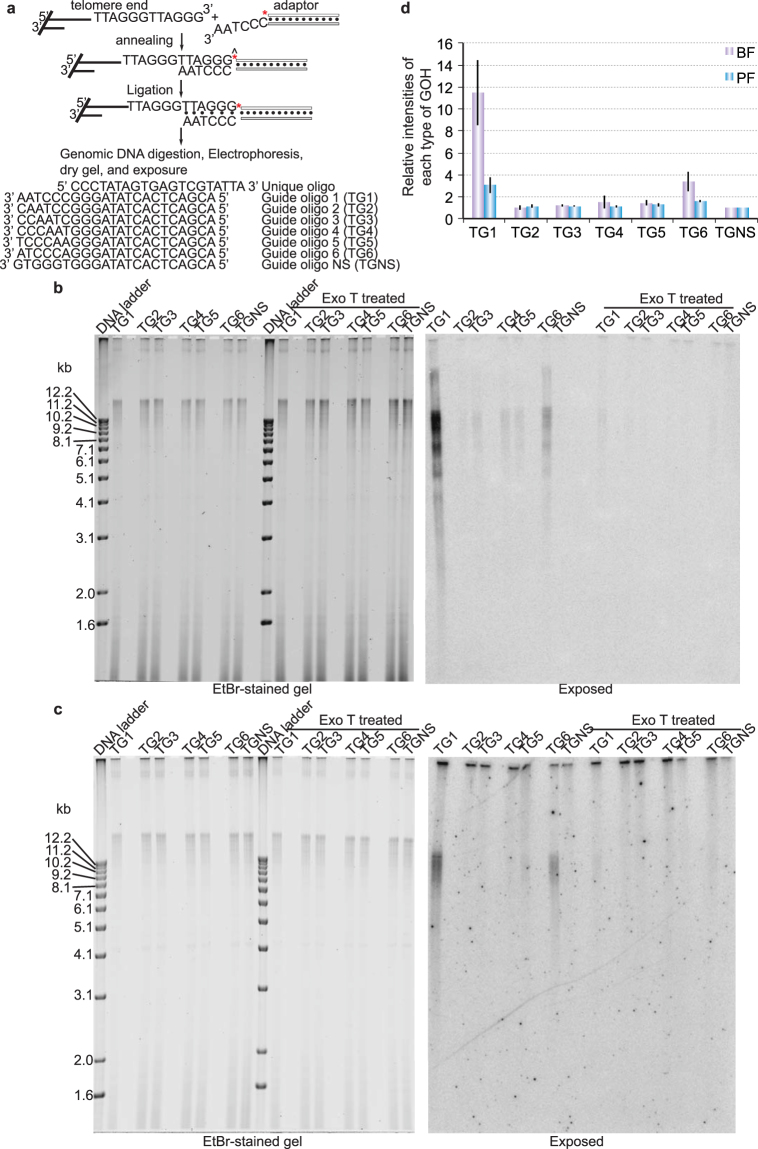
Most telomeres in *T*. *brucei* WT cells have a 3′ overhang that ends in 5′ TTAGGG 3′. (**a**) A diagram showing the principle of the adaptor ligation assay. Top, a natural chromosome end with a 3′ overhang (left) is ligated with an adaptor (right). The light bar of the adaptor represents the unique oligo and is radiolabeled at its 5′ end. The darker bar of the adaptor represents the common region of all guide oligos. Only when the adaptor matches the telomere end perfectly can the adaptor be ligated to the chromosome end and eventually migrate with the long telomere fragment in agarose gel electrophoresis. Bottom, the sequence of the unique oligo and seven different guide oligos are shown. The *T*. *brucei* genomic DNA from BF (**b**) and PF (**c**) WT cells (either treated with or without Exo T) was ligated to radioisotope-labeled different adaptors (TG1–6 and TGNS, as indicated on top of each lane), digested with AluI and MboI, and separated by agarose gel electrophoresis. The ethidium bromide-stained gel is shown on the left and the image of the gel exposed to a phosphorimager (exposed) is shown on the right. 1 kb DNA ladder (ThermoFisher) was used as a molecular weight marker. (**d**) Quantification of the adaptor ligation results in both BF and PF WT cells. Average values are calculated from five (BF) or three (PF) independent experiments. Error bars represent standard deviation.

*T*. *brucei* telomere DNA has a TTAGGG repeat sequence; therefore, telomere G-overhangs are expected to end in one of the six permutations of the 5′ TTAGGG 3′ sequence (5′ TTAGGG 3′, 5′ GTTAGG 3′, 5′ GGTTAG 3′, 5′ GGGTTA 3′, 5′ AGGGTT 3′, or 5′ TAGGGT 3′). Because each telomere overhang can only ligate with an adaptor having a compatible 3′ overhang (Fig. 1a), we can determine the telomere 3′ end sequence using the adaptor ligation assay (Fig. 1a). We have now optimized the adaptor ligation assay and carefully examined the telomere terminal structure in the bloodstream form (BF) and the procyclic form (PF) of *T*. *brucei* cells, which proliferate in the bloodstream of the mammalian host and the mid-gut of the insect vector, respectively. We were able to detect all six telomere G-overhang signals (Fig. 1b & c) in WT *T*. *brucei* cells, yet the signal intensities generated from each adaptor varied greatly. To better estimate the relative abundance of different telomere G-overhangs, we first calculated the raw G-overhang level: signal intensity of each adaptor ligation product from the exposed image was divided by the signal intensity of the same sample from the ethidium bromide stained gel. This corrected for small variations in the amount of loaded samples. We then calculated the final relative G-overhang level: each raw G-overhang signal for adaptors TG1–6 was normalized to that of the Non-Specific adaptor (TGNS) on the same gel, and the final G-overhang value for TGNS was arbitrarily set to 1. In BF cells, we found that the majority (77.5%) of telomeres can ligate to TG1 adaptor and therefore have a G-overhang that ends in 5′ TTAGGG 3′, while a smaller yet significant portion (22.5%) can ligate to TG6 adaptor and hence have an overhang that ends in 5′ TAGGGT 3′ (Fig. 1b). These two types of overhangs have significantly higher signal intensities (11 and 3, respectively) than the background (generated from the TGNS adaptor) (Fig. 1b & d). The remaining four overhang sequences generated signal intensities similar to background (1–1.5), indicating that these overhangs are rare, if any, in the cell. Unpaired two-tailed *t* tests indicate that TG2–TG5 signal levels are comparable (P > 0.05), while TG1 and TG6 levels are both significantly different from those of TG2–TG5 (P < 0.01). Additionally, TG1 and TG6 levels are also significantly different from each other (P < 0.01). Similarly, we detected that most telomere G-overhangs in PF cells also end in 5′ TTAGGG 3′ (65.3%) and some in 5′ TAGGGT 3′ (34.7%) (Fig. 1c). However, the relative intensities of the 5′ TTAGGG 3′-ending and 5′ TAGGGT 3′-ending overhangs (3 and 1.6, respectively) were both lower than their corresponding species in BF cells (Fig. 1d). The remaining four types of overhangs were again detected at levels (1.10–1.25) similar to that of the background. Unpaired two-tailed *t* tests indicate that TG2–TG5 signal levels are comparable (P > 0.05), while TG1 and TG6 levels are both significantly different from TG2–TG5 signals (P < 0.05). TG1 and TG6 levels are also insignificantly different from each other (P = 0.07) in PF cells.

Exo T is a single-stranded specific RNA or DNA nuclease that requires a free 3′ terminus and removes nucleotides in the 3′–5′ direction. In both BF and PF cells, treating genomic DNA with Exo T prevented ligation of the chromosome ends with the adaptors (Fig. 1b & c), confirming that the signals we detected were indeed due to a 3′ telomere overhang structure. Therefore, both infectious and insect stage *T*. *brucei* cells have similar telomere 3′ G-overhang structure at their chromosome ends. At both life cycle stages, most telomeres have 5′ TTAGGG 3′ends while a small portion of telomeres have 5′ TAGGGT 3′ ends. Because telomeres of BF and PF cells had similar G-overhang structures, we only examined mutant phenotypes in BF cells from here on.

### Depletion of *Tb*TRF decreases the amount of telomere G-overhangs

**Figure 2:**
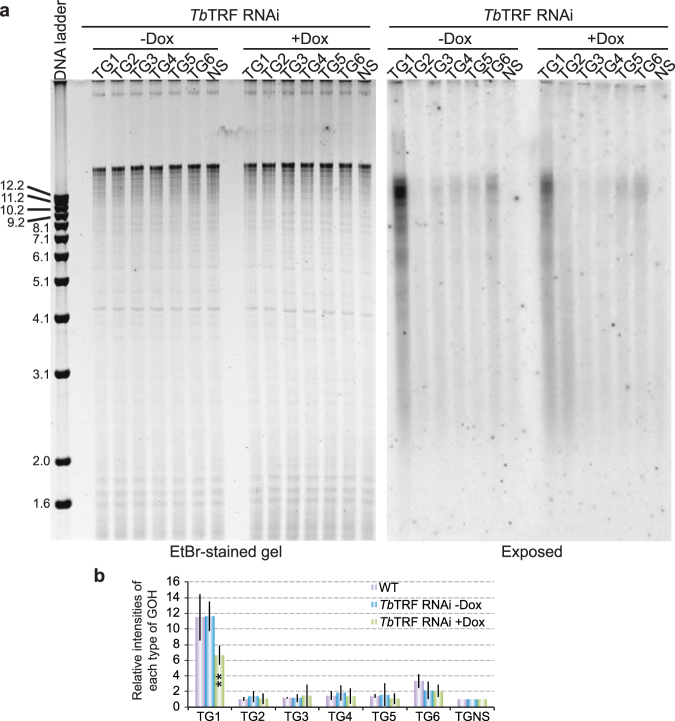
Depletion of *Tb*TRF leads to a decrease in the 5′ TTAGGG 3′-ending telomere G-overhangs. (**a**) The adaptor ligation assay was performed using genomic DNA isolated from the BF *Tb*TRF RNAi cells^51^ before (−Dox) and 24 hrs after the induction of *Tb*TRF RNAi (+Dox). The ethidium bromide-stained gel is shown on the left and the exposed image is shown on the right. (**b**) Quantification of the adaptor ligation results. Average values are calculated form five independent experiments. Error bars represent standard deviation. TG1–TG6 signals from induced cells were compared to those from uninduced cells by paired two-tailed *t* tests. **P < 0.01. P values greater than 0.05 are not indicated.

We previously identified *Tb*TRF as the duplex telomere DNA binding factor *in T*. *brucei* ^51^. Depletion of *Tb*TRF leads to a significant decrease in telomere G-overhang signals in the native in-gel hybridization assay^51^. Using the adaptor ligation assay, we detected a similar effect of *Tb*TRF. Genomic DNAs were isolated from *Tb*TRF RNAi cells^51^ before and after induction of RNAi for 24 hrs, and we were able to detect the telomere G-overhang from both samples (Fig. 2a). However, depletion of *Tb*TRF significantly decreased the abundance of 5′ TTAGGG 3′-ending telomere G-overhangs (average decrease of 43%, the paired two-tailed *t* test P value is 0.002) compared to uninduced cells and WT cells (Fig. 2b). This is consistent with our previous observations in the native in-gel hybridization, where induction of *Tb*TRF RNAi for 24 hrs resulted in a nearly 60% of decrease in the telomere overhang level^51^. In contrast, the 5′ TAGGGT 3′-ending telomere overhang levels appeared to decrease slightly upon depletion of *Tb*TRF (Fig. 2), but a careful statistical analysis indicated that this change was not significant (the paired two-tailed *t* test P value is 0.908). Our observations confirmed that the adaptor ligation assay and the native in-gel hybridization are both useful for examination of the telomere G-overhang structure in *T*. *brucei*. However, only the adaptor ligation assay yields information about the last nucleotide of the telomere 3′ end, allowing higher resolution analysis and revealing more details about the telomere end structure.

### Telomerase null *T. brucei* cells lost the 5′ TTAGGG 3′-ending telomere G-overhang

Telomerase-mediated *de novo* synthesis of the G-rich strand of telomeric DNA is expected to result in longer telomere G-overhangs. 5′ end resection by exonucleases can also elongate the telomere G-overhang in a telomerase-independent manner. Whether *T*. *brucei* telomerase is a major determinant of the telomere G-overhang level is unknown. We therefore examined the telomere overhang structure using the adaptor ligation assay in telomerase null cells.

**Figure 3:**
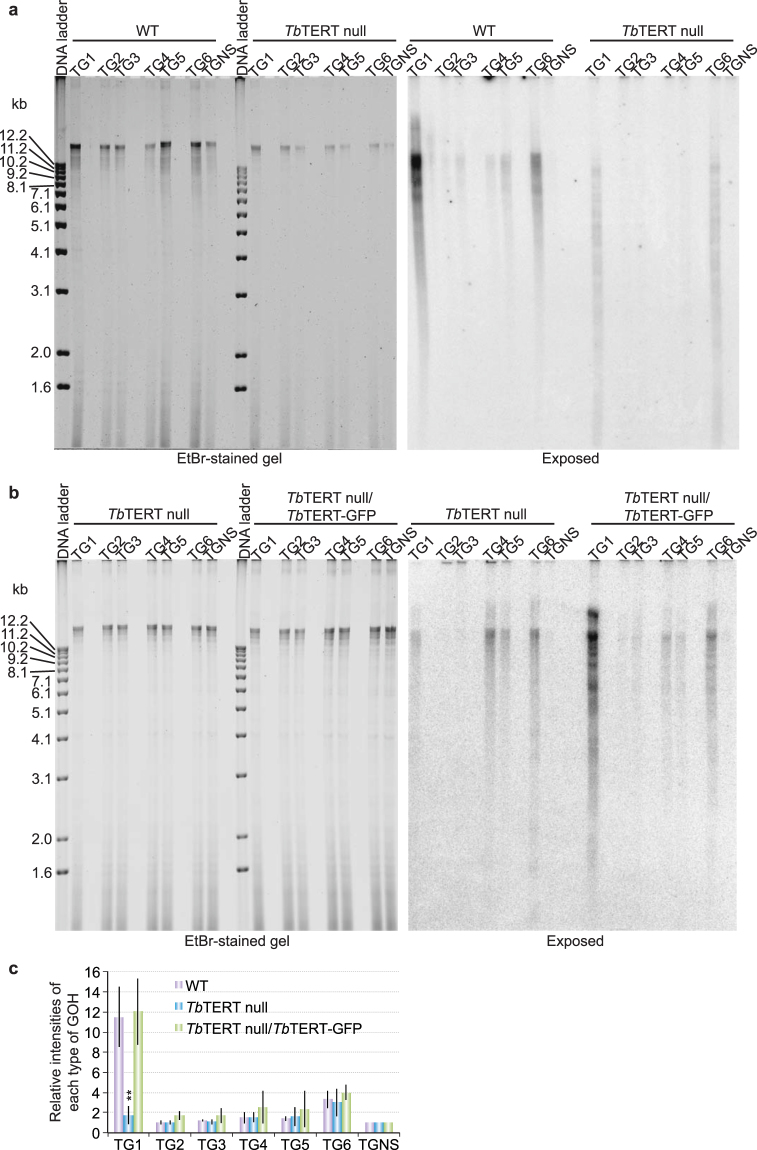
*Tb*TERT is necessary for normal levels of 5′ TTAGGG 3′-ending telomere G-overhangs. The adaptor ligation assay was performed using genomic DNA isolated from WT and *Tb*TERT null cells (**a**) or from *Tb*TERT null cells and *Tb*TERT null cells that also express an ectopic *Tb*TERT-GFP allele (**b**). The ethidium bromide-stained gels are shown on the left and the exposed images are shown on the right. (**c**) Quantification of the adaptor ligation results in *Tb*TERT null cells and *Tb*TERT null cells that express an ectopic *Tb*TERT-GFP allele in comparison to that in WT cells. Average values are calculated from six (*Tb*TERT null) or four (*Tb*TERT null + *Tb*TERT-GFP) independent experiments. Error bars represent standard deviation. The overhang signals from *Tb*TERT null cells were compared to those in WT cells by unpaired two-tailed *t* tests. **P < 0.01. P values greater than 0.05 are not indicated.

Telomerase has a catalytic protein subunit (TERT) and an intrinsic RNA (TR) that provides a short template for telomere DNA synthesis^54^. We first examined the telomere overhang structure in *Tb*TERT null cells that had been cultured for several months following deletion of the *TbTERT* gene^16^. The abundance of 5′ TTAGGG 3′-ending telomere overhangs decreased dramatically in *Tb*TERT null cells (Fig. 3). This defect was complemented by expressing an ectopic allele encoding C-terminally GFP-tagged *Tb*TERT (Fig. 3b & c) known to complement the telomere shortening phenotype of *Tb*TERT null cells^16^. Interestingly, the 5′ TAGGGT 3′-ending telomere overhangs appeared unaffected by the loss of telomerase (Fig. 3).

**Figure 4:**
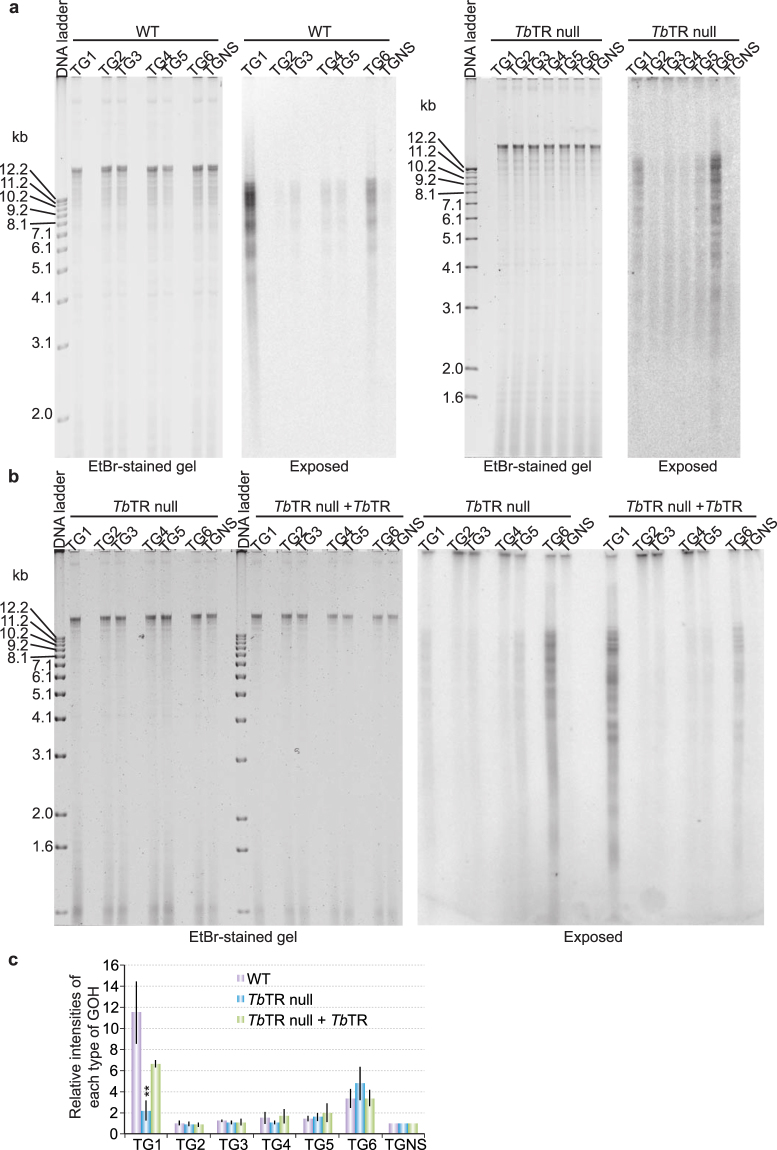
*Tb*TR is necessary for normal levels of 5′ TTAGGG 3′-ending telomere G-overhangs. The adaptor ligation assay was performed using genomic DNA isolated from WT (left panel) and *Tb*TR null cells (right panel) (**a**) or from *Tb*TR null cells and *Tb*TR null cells that also express an ectopic *Tb*TR allele (**b**). The ethidium bromide-stained gels are shown on the left and the exposed images are shown on the right in each panel. (**c**) Quantification of the adaptor ligation results in *Tb*TR null cells and *Tb*TR null cells that express an ectopic *Tb*TR allele in comparison to that in WT cells. Average values are calculated from eleven (*Tb*TR null) or three (*Tb*TR null + *Tb*TR) independent experiments. Error bars represent standard deviation. The overhang signals from *Tb*TR null cells were compared to those in WT cells by unpaired two-tailed *t* tests. **P < 0.01. P values greater than 0.05 are not indicated.

Similarly, in *Tb*TR null cells^17^, the 5′ TTAGGG 3′-ending telomere overhang signals decreased dramatically, while the 5′ TAGGGT 3′-ending overhang signals did not (Fig. 4). The decrease in 5′ TTAGGG 3′ signal was complemented by expressing an ectopic WT *TbTR* allele (Fig. 4b & c) previously shown to complement the telomere shortening phenotype of *Tb*TR null cells^17^. We analyzed the telomere G-overhang structure after culturing cells for 4 weeks or 11 weeks after deletion of the *TbTR* gene and did not observe significant differences in the telomere G-overhang structure (Fig. S1).

We conclude that, telomerase plays a critical role in maintaining the telomere G-overhang structure in *T*. *brucei*, and the 5′ TTAGGG 3′-ending telomere overhang is particularly dependent on telomerase.

### *Tb*Ku is required for normal levels of telomere G-overhangs

*Tb*Ku is the only non-telomerase protein identified so far in *T*. *brucei* that is required for telomere length maintenance^50^. Telomere shortening rate is 3–5 bp/population doubling (PD) in *Tb*Ku80 null cells^50^ and 3–6 bp/PD in *Tb*TERT or *Tb*TR null cells^16,17^.

**Figure 5:**
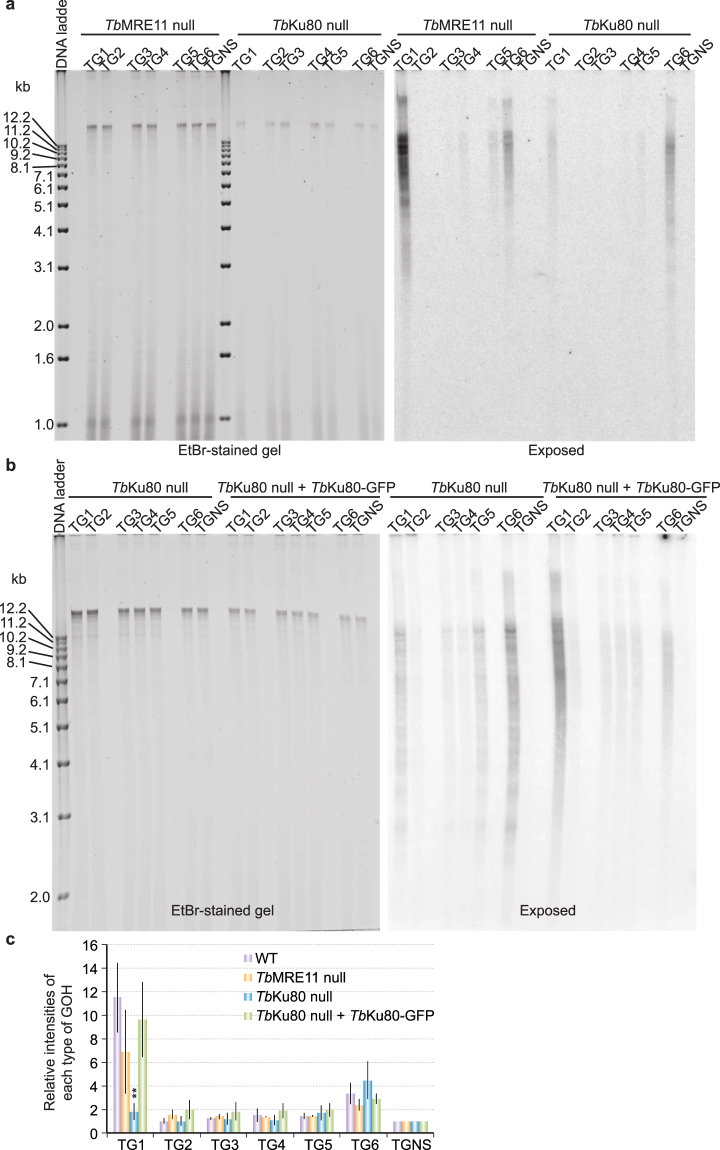
*Tb*Ku but not *Tb*MRE11 is necessary for normal levels of 5′ TTAGGG 3′-ending telomere G-overhangs. The adaptor ligation assay was performed using genomic DNA isolated from BF *Tb*MRE11 null^55^ and *Tb*Ku80 null cells (**a**) or from *Tb*Ku80 null cells and *Tb*Ku80 null cells that also express an ectopic *Tb*Ku80-GFP allele (**b**). The ethidium bromide-stained gels are shown on the left and the exposed images are shown on the right. (**c**) Quantification of the adaptor ligation results in *Tb*MRE11 null cells, *Tb*Ku80 null cells, and *Tb*Ku80 null cells that express an ectopic *Tb*Ku80-GFP allele in comparison to that in WT cells. Average values are calculated from three (*Tb*MRE11 null), five (*Tb*Ku80 null), or three (*Tb*Ku80 null + *Tb*Ku80-GFP) independent experiments. Error bars represent standard deviation. The overhang signals from *Tb*Ku80 null and *Tb*MRE11 null cells were compared to those in WT cells by unpaired two-tailed *t* tests. **P < 0.01. P values greater than 0.05 are not indicated.

Interestingly, loss of *Tb*Ku80 also resulted in the loss of the 5′ TTAGGG 3′-ending telomere overhang signals (Fig. 5). This phenotype was complemented by expressing an ectopic, C-terminally GFP-tagged *Tb*Ku80 known to complement the telomere shortening phenotype of *Tb*Ku80 null cells^50^ (Fig. 5b & c). The level of 5′ TAGGGT 3′-ending G-overhangs did not decrease in *Tb*Ku80 null cells (Fig. 5). In fact, the 5′ TAGGGT 3′-ending telomere overhang signals appeared to be subtly increased in *Tb*Ku80 and *Tb*TR null cells (Figs 4 and 5), although a careful analysis indicated that the difference was not statistically significant (when compared to WT cells, the unpaired two-tailed *t* test P values are 0.21 and 0.14 for *Tb*Ku80 null and *Tb*TR null cells, respectively).

Both telomerase and Ku null cells are defective in maintaining telomere length in *T*. *brucei*, and both lose the 5′ TTAGGG 3′-ending telomere G-overhang, suggesting that this specific overhang is formed during telomerase-mediated telomere elongation. To help test this hypothesis, we examined the telomere overhang structure in *Tb*MRE11 null cells^55^. MRE11 is an exonuclease and a component of the yeast MRE11/RAD50/XRS2 (MRX) or the human MRE11/RAD50/NBS1 (MRN) complex, which is critical for DNA end processing and plays an important role in DNA damage repair^56^. *S*. *cerevisiae* MRE11 appears to help recruit telomerase to the telomere^57^ and plays an important role in maintaining the telomere G-overhang structure^42^. Human MRE11 has similar functions^58,59^. However, *T*. *brucei* MRE11 is not required for telomere maintenance, although *Tb*MRE11 null cells are more sensitive to DNA damaging agents than WT cells^55,60^. We found that in *Tb*MRE11 null cells, the abundance of both the 5′ TTAGGG 3′-ending and the 5′ TAGGGT 3′-ending telomere overhangs appeared slightly lower than in WT cells (Fig. 5a). However, a careful quantification indicated that the differences are not statistically significant: the unpaired two-tailed *t* test P values were 0.11 and 0.21 for signals generated from TG1 and TG6 adaptors, respectively (Fig. 5c). Therefore, among the mutants we tested, *Tb*TERT null, *Tb*TR null, and *Tb*Ku80 null cells all have similar defects in telomere length maintenance and lose their 5′ TTAGGG 3′-ending telomere overhangs but not their 5′ TAGGGT 3′-ending telomere overhangs. In contrast, telomere elongation is normal in MRE11 null cells, and the amount of the telomere overhangs in these cells appears not significantly different from that in WT cells.

### The 5′ end structure of the *T. brucei* telomere

The 5′ end sequence at the telomere is another important feature of the telomere terminal structure and has not been determined in *T*. *brucei*. It is not expected to be affected by telomerase-mediated G-strand elongation; rather, it can be influenced by 5′ fill-in by DNA polymerase following elongation of the 3′ end by telomerase, and it can be influenced by exonucleases that process the 5′ end^3^. In addition, single-stranded telomere DNA binding proteins can also help specify the terminal nucleotide on the 5′ end^3^.

**Figure 6:**
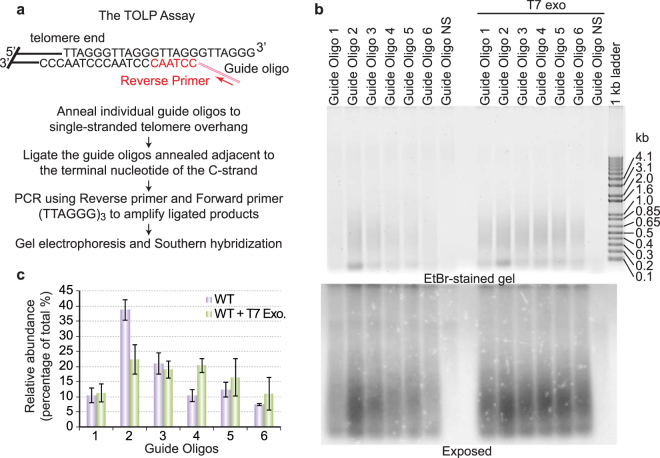
Telomeres in *T*. *brucei* cells prefer to have a 5′ end with a sequence of 5′ CCTAAC 3′. (**a**) A diagram showing the principle of the Telomere Oligo Ligation-mediated PCR (TOLP) assay. Seven different telomere guide oligos are separately ligated to intact genomic DNA. Only when the oligo is aligned immediately next to the 5′ end of the telomere can the oligo be ligated to the chromosome DNA. Subsequently the ligation products are amplified by PCR using a forward primer with the sequence of (TTAGGG)_3_ and a backward primer with the sequence identical to the common regions of all guide oligos. The PCR products were then separated by agarose gel electrophoresis followed by Southern analysis using a telomeric probe. (**b**) TOLP was performed using genomic DNA isolated from WT BF cells that was treated with or without T7 exonuclease. The ethidium bromide-stained gel is shown on the top, and the Southern hybridization result using a telomeric probe is shown on the bottom. (**c**) Quantification of the TOLP signals from different guide oligos is shown. Average is calculated from three independent experiments. Error bars represent standard deviation.

We determined the telomere 5′ end sequences using a Telomere Oligo Ligation-mediated PCR (TOLP) assay (Fig. 6a), which we developed from the published single telomere length assay (STELA)^61–63^. Although any guide oligo can anneal to the single stranded telomere 3′ overhang at multiple positions due the repetitiveness of telomere DNA, a guide oligo can be ligated to the chromosome DNA only when it is annealed adjacent to the terminal nucleotide of the C-strand (Fig. 6a). Subsequent PCR amplification relies on the presence of the unique sequence at the guide oligo tail and can only amplify ligated products.

Using the TOLP assay and the guide oligo NS as a background control, we found that in WT BF *T*. *brucei* cells, guide oligo 2 can be efficiently ligated onto telomere ends, yielding a signal that is significantly higher than that obtained with any other guide oligo (Fig. 6b & c). Therefore, most telomeres (~40% of all signals, Fig. 6c) have a 5′ end sequence of 5′ CCTAAC 3′. Guide oligo 3 ligated products were next in abundance (~20%, Fig. 6b & c), representing a 5′ end sequence of 5′ CTAACC 3′. Signals resulting from guide oligos 1, 4, 5, and 6 ligations were comparably low, each representing ~10% of total signal detected (Fig. 6). As a control, we treated the genomic DNA with T7 exonuclease before ligating with the guide oligos. T7 exonuclease processively removes the 5′ mononucleotide from duplex DNA in the 5′ to 3′ direction. Treating the genomic DNA with T7 exonuclease is expected to generate telomere 5′ ends bearing random permutated telomere sequences. Indeed, after T7 exonuclease treatment, guide oligos 2, 3, 4, and 5 ligation products were detected at similar levels (~20% of total signal each), while guide oligo 1 and 6 ligation products were detected at slightly lower levels (~10% of total signal) (Fig. 6b & c). Therefore, the nucleotide sequence at *T*. *brucei* telomere 5′ end is not random, and the 5′ CCTAAC 3′ sequence is preferred.

## Discussions

The telomere G-overhang structure is essential for telomere maintenance and chromosome end protection^3^. We previously detected the telomere G-overhang structure in *T*. *brucei* using in-gel hybridization^51^ and adaptor ligation assays^18^. Now, after optimizing the adaptor ligation assay, we verified that *T*. *brucei* telomeres end in a G-overhang structure.

We have further determined the 3′ end telomere sequences and showed that *T*. *brucei* has two types of telomere G-overhang. The predominant overhang ends in 5′ TTAGGG 3′ and the other in 5′ TAGGGT 3′. Using a vector-adaptor ligation protocol to clone the terminal telomere sequences into plasmids^19,64–66^, telomeres carrying the terminal 5′ TTAGGG 3′ sequence were identified in *Trypanosoma brucei* ^19^ and *Trypanosoma cruzi* ^64^, while telomeres bearing the terminal 5′ TAGGGT 3′ sequence were cloned from *Leishmania major* ^66^ and *Leishmania donovani* ^65^. Interestingly, we detected both 5′ TTAGGG 3′-ending and 5′ TAGGGT 3′-ending telomeres in *T*. *brucei* using the adaptor ligation method, indicating that our assay is more sensitive and can detect less abundant ends. We have also determined the percentage of telomeres bearing each type of G-overhang, while the vector-adaptor ligation assay^19,64–66^ cannot show distribution of different types of telomeres. Whether *T*. *cruzi* and *Leishmania* have more than one type of telomere 3′ end is unknown, and the optimized adaptor ligation assay would be a useful tool for answering this question. Intriguingly, the cloning of telomeres ending in 5′ TAGGGT 3′ from *Leishmania* cells using the vector-adaptor ligation approach^65,66^ suggests that this type of telomere is abundant in *Leishmania*, which is different from the scenario in *T*. *brucei* and *T*. *cruzi*, even though these organisms are closely related. The *Leishmania* telomerase RNA gene has recently been identified and its template shown as 5′ ACCCTAACCCTA 3′^67^. The extra A residue at the 5′ end of the *Leishmania* TR template is not present in the *T*. *brucei* TR template 5′ CCCTAACCCTA 3′^17^, which would explain the difference in telomere 3′ ends among these *Kinetoplastids*.

In addition to the telomerase-mediated telomere elongation, multiple other processes can influence telomere G-overhang length and the terminal sequences^3^. Therefore, telomerase activity is necessary for the normal length of telomere G-overhang in certain eukaryotic organisms^33,34^ but not others^35–37^. Interestingly, we found that *T*. *brucei* telomerase is a major factor contributing to the generation/maintenance of telomere G-overhangs ending in 5′ TTAGGG 3′. In addition to *Tb*TERT and *Tb*TR null cells, *Tb*Ku null cells exhibited the same phenotype: the 5′ TTAGGG 3′-ending G-overhang was depleted. Since normal telomere maintenance is abolished in *Tb*Ku null cells^50^, our observations strongly suggest that the 5′ TTAGGG 3′-ending G-overhangs are generated by telomerase-mediated telomere synthesis.

Telomerase synthesizes the telomere G-strand DNA through repetitive cycles of copying the *Tb*TR template followed by a translocation step^68^. The G-overhang 3′ end sequence is expected to be 5′ TTAGGG 3′ right after synthesis of each telomere repeat according to the *Tb*TR template, since we have determined the template of *Tb*TR to be 5′ CCCTAACCCTA 3′^17^. This expectation has now been confirmed by our adaptor ligation assay, suggesting that after *de novo* synthesis of the telomeric DNA by telomerase, the 3′ end of the G-overhang is mostly unprocessed. This is a much simpler scenario than that in many other eukaryotic cells, where multiple factors are involved in telomere end processing^3^. It is interesting to note that the telomerase activity also appears to be unchecked in *T*. *brucei*, as continuous telomere elongation at a rate of 6–10 bp/PD has been observed in multiplying *T*. *brucei* cells^17,69^. Most telomeres are only occasionally shortened except for the active ES-adjacent telomere, which is often subjected to large stochastic truncations^69,70^ likely because the active ES-adjacent telomere is transcribed^30^. Therefore, telomerase-mediated telomere elongation appears to be an unregulated process in *T*. *brucei*, unlike in yeasts, worms, mammals, and plants^71,72^. These observations suggest that telomere synthesis and end processing is very simple in *T*. *brucei*, making *T*. *brucei* an ideal model system to study telomerase action. In addition, the telomere G-overhang status can directly reflect whether telomerase is active at the telomere end and can thus be used as an indicator of *in vivo* telomerase activity at the telomere.

We found that a small percentage of the *T*. *brucei* telomere G-overhangs end in 5′ TAGGGT 3′. This could be explained if the telomerase has a tendency to dissociate with its substrate prematurely after extending one T residue. However, the abundance of 5′ TAGGGT 3′-ending telomeres appears to be unchanged or slightly increased rather than decreased in telomerase null cells, arguing against this hypothesis. Alternatively, certain single-stranded telomere DNA binding factors may mask the 5′ TAGGGT 3′ sequence while leaving the last 5′ TAGGG 3′ residues exposed for nuclease degradation after the telomerase extension step. So far, we have not identified any telomere-specific single-stranded DNA binding factors. However, RPA1 is likely to bind the telomere G-overhang, as its homologue in *Leishmania* colocalizes with telomeres *in vivo* ^73^. Finally, an enzyme with a specialized terminal nucleotidyltransferase (TdT) activity may occasionally add a T residue to telomere ends after their extension by telomerase. For example, the human DNA polymerase λ has a TdT activity^74^ that preferentially adds one or two pyrimidine nucleotides and requires a single-stranded 3′ overhang of 9–12 nt for optimal efficiency^75,76^. Such activity would be able to generate telomeres that end in 5′ TAGGGT 3′. However, the *T*. *brucei* genome does not appear to encode a homolog of nuclear DNA polymerase λ.

Using the TOLP analysis, we found that the 5′ end of the *T*. *brucei* telomere G-overhang has a preference for the sequence 5′ CCTAAC 3′, filling the knowledge gap about this important feature of the telomere terminal structure. Single stranded telomere DNA binding factors that bind the telomere G-overhang, such as human POT1, are important for dictating the telomere 5′ end sequence^77^. It is possible that *T*. *brucei* also has a telomere G-overhang binding factor that controls 5′ end processing. Identification of the telomere protein that specifically binds the telomere 3′ G-overhang will further allow us to test this hypothesis. Alternatively, in lagging strand DNA synthesis, the last RNA primer may be located at a relatively defined position with respect to the 3′ end of the template strand. Subsequent removal of the last primer would then leave a telomere 5′ end that has preferred sequence.

In this study, we have shown that the adaptor ligation and TOLP assays are useful tools to examine the telomere terminal structure with high resolution. Importantly, both the 5′ and 3′ ends of *T*. *brucei* telomeres have preferred ending sequences. Our observations strongly suggest that telomerase mediated telomere length maintenance is a key determining factor of the 3′ end telomere sequences and that telomerase products are not extensively processed. Therefore, our study not only reveals the detailed structure of the telomere G-overhang but also sheds light on better understanding of the telomerase action in this important human pathogen.

## Materials and Methods

### The adaptor ligation assay

The adaptor ligation assay was modified from Jacob *et al*.^53^. Seven different guide oligos (guide oligos 1–6 and NS) were separately annealed to the ^32^P end labeled unique oligo to generate seven different adaptors (TG1–6 and TGNS adaptors), which were then ligated onto genomic DNA. Subsequently, the genomic DNA was digested with AluI and MboI (New England Biolabs) followed by agarose gel electrophoresis. The Ethidium Bromide-stained agarose gel was scanned using a Typhoon 9410 scanner, where the signal served as a loading control. Subsequently, the gel was dried and exposed to a phosphorimager screen followed by scanning using the Typhoon 9410 scanner. ImageQuant was used to quantify the radioactive signal and the ethidium bromide-staining signal. The radioactive signal for each adaptor ligated sample was first normalized against the corresponding ethidium bromide-staining signal then divided by the signal from the TGNS adaptor ligated sample. For the EXO-T control, approximately 40 µg of genomic DNA was treated with 50 U of EXO-T (New England Biolabs) for 16 hours. DNA was purified by phenol/chloroform extraction followed by precipitation.

### The TOLP assay to examine the telomere 5′ end sequence

TOLP was modified from STELA^61–63^. Briefly, 100 ng genomic DNA was ligated to each guide oligo (10 pmole) using T4 DNA ligase (ThermoFisher). One fifth of the ligation mixture was used for PCR using the forward primer (5′ TTAGGGTTAGGGTTAGGG 3′) and the backward primer (5′ ACGACTCACTATAGGG 3′); the sequence of the latter oligo is identical to the common region of all guide oligos. The PCR products were subsequently separated by agarose gel electrophoresis followed by Southern blotting using a telomeric probe. Southern blots were exposed to Phosphorimager screens, and ImageQuant was used to quantify the hybridization signals. The signal from the NS guide oligo was used to normalize all other signals. For T7 EXO nuclease control, 1.3 μg of genomic DNA was incubated with 10 U of T7 exo (New England Biolabs) for 35 min at 25 °C.

### Plasmids

#### *Tb*Ku deletion constructs

The sequences immediately upstream of the *TbKu80* gene, the LoxP-flanked *Hygromycin resistance-Thymidine Kinase* fusion gene, and the sequence immediately downstream of the *TbKu80* gene are PCR amplified and inserted into pBluescript SK in this order to make the *Tb*Ku80 knockout construct with the *HYG-TK* marker. A separate *Tb*Ku80 knockout construct with the *Puromycin resistance* (*PUR*) marker was similarly generated.

### *T. brucei* strains

All *T*. *brucei* strains used in this study are derived from bloodstream form Lister 427 cells that express VSG2 and express a T7 polymerase and Tet repressor (SM)^78^ or procyclic form Lister 427 WT cells. SM cells were sequentially transfected with the two *Tb*Ku80 knockout constructs to establish the *Tb*Ku80 null cells, whose genotype was confirmed by Southern blotting. The *Tb*Ku80 null cells were transfected with the pLEW82-eGFP-Ku80-GFP inducible expression construct^50^ to obtain *Tb*Ku80 null + *Tb*Ku80-GFP complementation strain. All bloodstream form *T*. *brucei* cells were cultured in HMI-9 medium supplemented with 10% FBS and appropriate antibiotics. All procyclic form *T*. *brucei* cells were cultured in SDM79 medium supplemented with 10% FBS and appropriate antibiotics.

### Statistical Analysis

Statistical analyses were done in MS Excel. For *Tb*TRF RNAi cells, before and after + Dox data (TG1–6) were compared by paired two-tailed *t* tests. Within WT cells, signals from different adaptors (TG1–TG6) were compared with each other by unpaired two-tailed *t* tests. All mutant cells were compared with the WT cells for corresponding telomere overhang signals (TG1–TG6) by unpaired two-tailed *t* tests. For all unpaired two-tailed *t* tests, we first performed the F test to determine whether the variances are equal, so that the correct parameter is set when performing the *t* tests. The *t* test P values are stated in the text or indicated in figures. *P < 0.05; **P < 0.01; ***P < 0.001.

All data generated or analyzed during this study are included in this published article (and its Supplementary Information files).

## Electronic supplementary material

Supplemental information
