# Supplementary material for: Telomerase activity is required for the telomere G-overhang structure in Trypanosoma brucei
Source: Sci Rep. 2017 Nov 22;7:15983. doi: 10.1038/s41598-017-16182-y (PMC5700094; doi:10.1038/s41598-017-16182-y)

The telomerase activity is required for the telomere G-overhang structure in *Trypanosoma brucei*

Ranjodh Sandhu[1,5] and Bibo Li[1,2,3,4,*]

Supplemental Figure Legends

Figure S1. The telomere G-overhang level does not change significantly in *Tb*TR null cells that are cultured for 4 weeks or 11 weeks. The adaptor ligation assay was performed using genomic DNA isolated from *Tb*TR null cells at 4 weeks (left) or 11 weeks (right) after the *TbTR* gene was deleted. The ethidium bromide-stained gel is shown on the left and the exposed image is shown on the right.

Sandhu & Li
Supplemental figure 1

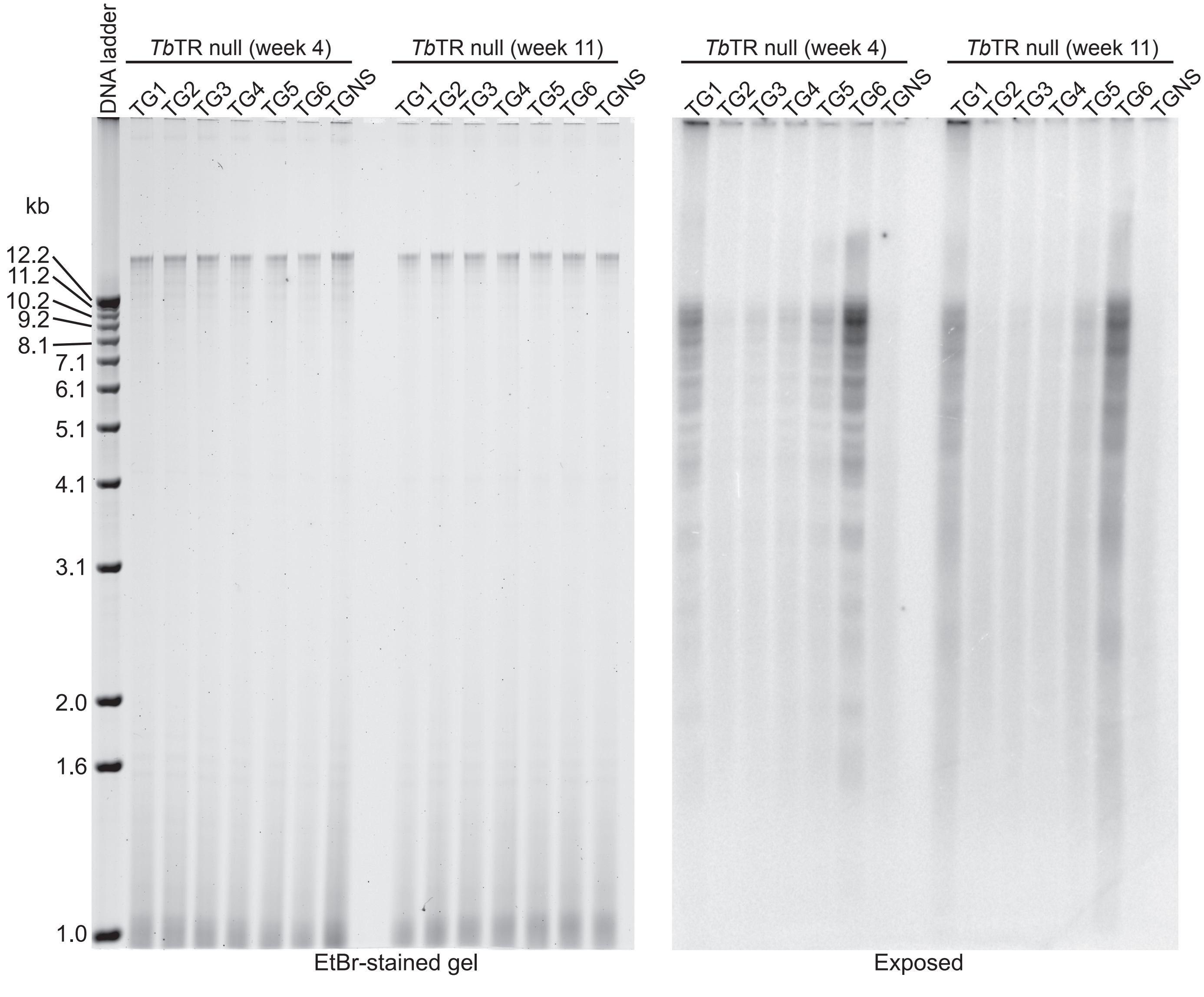

Supplement: Supplementary file 1 — Supplemental information [file 41598_2017_16182_MOESM1_ESM.pdf]
